# Chinese medicines as a resource for liver fibrosis treatment

**DOI:** 10.1186/1749-8546-4-16

**Published:** 2009-08-20

**Authors:** Yibin Feng, Kwok-Fan Cheung, Ning Wang, Ping Liu, Tadashi Nagamatsu, Yao Tong

**Affiliations:** 1 School of Chinese Medicine, The University of Hong Kong, 10 Sassoon Road, Pokfulam, Hong Kong, PR China; 2 Department of Medicine, The University of Hong Kong, 21 Sassoon Road, Pokfulam, Hong Kong, PR China; 3 Department of Cell Biology, Shanghai University of Traditional Chinese Medicine, Shanghai 201203, PR China; 4 Department of Pharmacobiology and Therapeutics, Faculty of Pharmacy, Meijo University, 150 Yagotoyama, Tenpakuku, Nagoya 468-8503, Japan

## Abstract

Liver fibrosis is a condition of abnormal proliferation of connective tissue due to various types of chronic liver injury often caused by viral infection and chemicals. Effective therapies against liver fibrosis are still limited. In this review, we focus on research on Chinese medicines against liver fibrosis in three categories, namely pure compounds, composite formulae and combination treatment using single compounds with composite formulae or conventional medicines. Action mechanisms of the anti-fibrosis Chinese medicines, clinical application, herbal adverse events and quality control are also reviewed. Evidence indicates that some Chinese medicines are clinically effective on liver fibrosis. Strict quality control such as research to identify and monitor the manufacturing of Chinese medicines enables reliable pharmacological, clinical and in-depth mechanism studies. Further experiments and clinical trials should be carried out on the platforms that conform to international standards.

## Background

Liver fibrosis is a condition of abnormal proliferation of connective tissue due to various types of chronic liver injury often caused by viral infection and chemicals. Hepatitis B viral (HBV) infection is the major cause of liver fibrosis in China, whereas hepatitis C viral (HCV) infection and alcohol are the main causes in the United States, Europe and Japan [1-4]. Liver fibrosis may progress into liver cirrhosis and other complications coupled with carcinogenesis [5,6]. The pathogenesis of liver fibrosis involves the activation of hepatic stellate cells (HSCs), the over-expression and over-secretion of collagens, and consequently an excessive accumulation of extracellular matrix (ECM) proteins [7]. Research has been focused on the management of liver fibrosis including the elimination of primary diseases, immunomodulation, suppression of hepatocyte inflammation, prevention of death and damage of hepatocytes, inhibition of over-secretion and accumulation of ECM proteins, promotion of ECM degradation, improvement of microcirculation and metabolism of liver and reduction of complications [8]. The reversal of liver fibrosis and even cirrhosis has been documented [9].

Complementary and alternative treatments of liver fibrosis have been under active research worldwide [10-12]. In Chinese medicine, liver fibrosis is thought to be caused by 'poor blood circulation, toxin stagnation and a deficiency of healthy energy' (dysregulated metabolism). Thus, Chinese medicine therapy to treat liver fibrosis is mainly based on reducing blood stagnation, resolving stasis, eliminating toxins and enhancing body immunity.

This review aims to provide an overview on the types of Chinese medicines used to treat liver fibrosis.

## Chinese medicines used to treat liver fibrosis

### Compounds and extracts

Around 20 compounds or extracts from Chinese medicines have been reported to have liver protective and anti-fibrotic effects. Various studies on their chemistry and pharmacology as well as clinical trials have been carried out to study these compounds or extracts. Table 1 summarizes those with liver protection and anti-fibrotic effects demonstrated in various research reports [13-68].

**Table 1:** Anti-fibrosis effect of compounds or extracts derived from Chinese medicines

Compounds or extracts and major references	Pharmacological actions and clinical indications	Botanic source
*Salvia miltiorrhiza* Extract & SA-B [13-18]	Reduce ALT and AST activities, inhibit protein expressions of TGF-β1, type I collagen and Smad3, anti-oxidation, down- regulate TGF-β1, TIMP-1 gene expression and MAPK activity, anti-nitric oxide, anti-apoptosis, apply to CHB patients	Root of *Salviae miltiorrhiza *Bge.
Glycyrrhizin [19-26]	Reduce ALT and AST activities, inhibit NF-κB binding activity, down-regulate smurf2 gene expression, apply to CHC patients and prevent hepatocarcinoma in patients with HCV-associated cirrhosis	Rhizome of *Glycyrrhiza uralensis *Fisch., *Glycyrrhiza inflata *Batal. or *Glycyrrhiza glabra *L.
Tetrandrine [27-32]	Down-regulate c-fos and c-jun gene expression, anti-nitric oxide, up-regulate Smad7 gene expression, apply to CHB patients, down-regulate NF-κB signalling cascade and biomarker such as ICAM-1 and α-SMA	Root of *Stephaniae tetrandrae *S. Moore
Matrine & Oxymatrine [33-35]	Inhibit PDGF and TGF-β1 actions, inhibit HBV-DNA, improve liver function in patients with CHB or CHC patients	Root of *Sophorae flavescentis *Ait
Taurine [36,37]	Inhibit TGF-β1 action, collagen formation in M cell culture system, reduce oxidative stress	*Calculus Bovis*
Tetramethylpyrazine (Chuanxiongzine) [38]	Anti-oxidation, synergic anti-hepatic fibrosis effect with rehin, apply to CHB patients	Rhizome of *Ligusticum chuanxiong *Hort.
Rehin, emodin [39-41]	Inhibit TGF-β1 expression, anti-HSC proliferation	Root and Rhizome of *Rheum palmatum *L., *Rheum tanguticum *Maxim. Ex Balf. or *Rheum officinale *Baill.
Curcumin [42,43]	Anti-oxidative effect, activate PPARgamma to reduce cell proliferation, induce apoptosis and suppress ECM gene expression *in vitro *and *in vivo*	Rhizome of *Curcumae longa *L.
*Panax Notoginseng *saponin and its water-extract [44-46]	Reduce AST and ALT, increase liver and serum SOD, reduce serum liver fibrosis markers levels, prevent liver fibrosis and hepatic microvascular dysfunction in liver fibrosis rats	Root of *Panax notoginseng *(Burk)F.H. Chen
Cordyceps polysaccharide [47,48]	Increase CD_4_/CD_8 _T lymphocytes ratio and decrease HA and PC III, inhibit TGF-β1 and PDGF expressions, reduce AST and ALT, apply to CHB patients	The complex of the stroma of the fungus *Cordceps sinensis *(berk.)Sacc. and larva of caterpillar on which the fungus grows
*Ginkgo biloba *extract [49,50]	Reduce ALT and AST, anti-oxidation, suppress NF-κB activation, inhibit TGF-β1 and collagen gene expression in rats	Leaves of *Ginkgo bioba *L.
Artemisinin/artesunate [51]	As inhibitors of hepatitis B virus production	Aerial part of *Artemesia annua *L.
Berberis aristata fruit extract and berberine [52-62]	Reduce AST and ALT, anti-oxidation, suppress expression of NF-κB, α-SMA, TGF-β1, anti-liver cancer, induce apoptosis in cancer cell lines and animal models	Rhizome of *Coptis chinensis *French., *Coptis teeta *Wall., *Coptis japonica *Makino., other genus *Berberis*
Aucubin [63,64]	Reduce AST and ALT, against HBV replication, suppress NF-κB activation in cell or animal models.	Ripe seed of *Plantago asiatica *L.
*Ganoderma lucidum *extract & Ganoderma polysaccharide [65,66]	Reduce AST, ALT, ALP, Tbil and the collagen content in rats with cirrhosis induced by biliary obstruction in rats, inhibit HSCs cells proliferation through blocking PDGFβR phosphorylation	*Ganoderma lucidum*
Gypenoside [67]	Inhibits HSCs proliferation, arrest HSC cells at G1 phase, inhibit the signal pathway of PDGF-Akt-p70 and down-regulate of cyclin D1 and D3 expression	*Gynostemma pentaphyllum*
*Solanum nigrum Linn *extract [68]	Reduce AST, ALT, ALP, Tbil, modulate GSTs and SOD, repress the production of free radicals	*Solanum nigrum *Linn

### Composite formulae

More than ten composite formulae for liver fibrosis have been reported [69-108]. Table 2 summarizes traditional composite formulae such as *Yinchenhao Tang, Xiao Chaihu Tang*, *Buzhong Yiqi Tang *and *Renshen Yangrong Tang *as well as modern formulae such as *Fufang Jinsane, Danshen Taoxiong Tang, Ershen Zezhu Tang, Buqi Jianzhong Tang*, *Fangji Tang*, *Handan Ganle*, *Ganzhifu *and *Fuzheng Huayu*.

**Table 2:** Anti-fibrosis effect of composite formulae

Composite formulae and major references	Pharmacological actions and clinical indications	Compositions of formulae
Yinchenhao Tang [69-78]	Induce HSCs apoptosis, inhibit HSCs activation, reduce collagen deposition and α-SMA and decrease the serum level of HA, apply to postoperative biliary atresia patients and icteric patients with cirrhosis	*Herba Artemisiae Scopariae*, *Radix et Rhizoma Rhei*, *Fructus Gardeniae*
*Xiao Chaihu Tang *[79-90]	Inhibit TGF-β1 and PDGF expressions, regulate MMPs/TIMPs balance, increase IL-12 production, suppress HSC activation, apply to CHC and CHB patients	*Radix Bupleuri*, *Radix Scutellariae*, *Rhizoma Pinelliae*, *Radix Ginseng*, *Fructus Jujubae*, *Radix Glycyrrhizae*
*Buzhong Yiqi Tang *[91,92]	Immunoregulation, inhibit TGF-β1 and IL-13 production, apply to CHC patients	*Radix Astragali, Radix Glycyrrhizae, Radix Ginseng, Radix Angelicae Sinensis, Pericarpium citri reticulatae, Rhizoma Cimicifugae, Radix Bupleuri, Rhizoma Atractylodis macrocephalae*
*Renshen Yangrong Tang *[92-94]	Immunoregulation, inhibit TGF-β1 and IL-13 production, apply to CHC patients	*Radix Astragali*, *Radix Angelicae sinensis*, *Cortex Cinnamomi*, *Radix Glycyrrhizae*, *Pericarpium citri reticulatae*, *Rhizoma Atractylodis macrocephalae*, *Radix Ginseng*, *Radix Paeoniae alba*, *Radix Rehmanniae*, *Fructus Schisandrae chinensis*, *Poria*, *Cortex et Radix Polygalae*
*Fufang Jinsan E *[95]	Inhibit TGF-β1 and Smad3, Up-regulate Smad7 in liver fibrotic rats	*Radix Curcumae*, *Rhizoma Sparganii, Rhizoma Curcumae*
*Denshen Taoxiong Tang *[96]	Anti-ascites, regulate urine sodium concentration in liver fibrotic mouse	*Radix Salviae Miltiorrhizae*, *Semen Persicae*, *Rhizoma Chuanxiong*
*Ershen Zezhu Tang *[96]	Anti-ascites, regulate urine sodium concentration in liver fibrotic mouse	*Radix Codonopsis*, *Radix Salviae miltiorrhizae*, *Rhizoma Atractylodis macrocephalae*, *Rhizoma Alismatis*
*Buqi Jianzhong Tang *[97,98]	Diuretic effect, increase excretion Na+, reduce GPT and GOT, apply to cirrhosis ascites	*Largehead Atractyloidis Rhizoma, Hoelen, Aurantii Nobilis Pericarpium, Radix Ginseng, Radix Scutellariae, Magnolia Bark, Alisma Rhizoma, Radix Ophiopogonis, Atractylodis Rhizoma*
*Fangji Tang *[97,98]	Diuretic effect, increase excretion Na+, reduce GPT and GOT, apply to cirrhosis ascites	*Sinomeni Claulis Et Rhizoma, Mori Contex, Hoelen* *Preilla Herba, Saussurae Radis*
*Handan Ganle *[99-102]	Anti-oxidatation, collagenolytic effect, regulate MMPs/TIMPs balance, apply to CHB patients	*Radix Sophorae Flavescentis*, *Radix Salviae miltiorrhizae*, *Radix Paeoniae*, *Radix Astragali*, *Folium Ginkgo*
*Ganzhifu *[103]	Anti-oxidation, reduce collagens, anti-liver fibrosis in liver fibrotic rats	*Rhizoma Zingiberis*, *Ramulus Cinnmomi*, *Radix Aconiti Lateralis preparata*, *Radix Astragali*, *Radix Bupleuri*, *Fructus Aurantii*, *Rhizoma Atractylodis macrocephalae*, *Radix Glycyrrhizae*
*Fuzheng Huay *[104-108]	Significantly decrease HA, LM, P-III-P and IV-C content, improve serum Alb, ALT, AST, GGT, LM, HA, Hyp and ration of BCAA/AAA in animals and CHB patients. Inhibit HSCs activation via FN/integrin signaling.	*Radix Salvia miltiorrhizae, Cordyceps mycelia extract, Semen Persicae, Gynostemma Pentaphyllammak, Pollen Pini, Fructus schisandrae chinensis*

### Combination therapy

Studies [109-118] show that combination therapy improves clinical anti-fibrotic effects by using a single compound with composite formulae or Chinese medicines with conventional medicines (Table 3).

**Table 3:** Anti-fibrosis effect of combinations of single compound and formulae or Chinese medicines and conventional medicines

Combination of drugs and major references	Clinical indications and pharmacological actions or side effects
ITF-α. injection + glycyrrhizin (Stronger Neo Minophagen C) injection [109]	CHC patients. With IFN therapy, ALT levels did not decrease more than 50%, while with IFN combined with SNMC therapy, ALT levels decreased approximately 70% in all patients (one became normal), but no other parameters were changed.
Ursodeoxycholic acid P.O + glycyrrhizin P.O [110]	CHC patients belong to interferon-resistant or unstable patients. Improving liver-specific enzyme abnormalities: AST, ALT and gamma-glutamyl transpeptidase, no change HCV-related factors or liver histology compared with control.
Matrine injection + *Xiao Chaihu Tang *P.O [111]	Liver fibrosis patients. Combination therapy improves AST, ALT and reduces HA, LN, CIV, TGF-β1 and TNF-α.
IFN-γ or IFN-α. injection + *Xiao Chaihu Tang *(*Sho-saiko-to*) P.O [112-115]	CHB patients. Combination therapy improves AST, ALT, Tbil and has synergistic anti-fibrosis in biochemical parameters, but IFN and/or Sho-saiko-to may also induce acute interstitial pneumonitis.
Tiopronin P.O + *Xiao Chaihu Tang *P.O [116]	CHB patients. Synergistic effects in improving liver functions and fibrotic factors.
Lamivudine + *Radix Salviae Miltiorrhizae *[117]	CHB patients. Treatment with both drugs was better than one and more effective than the control group in parameters of liver function and liver fibrosis.
*Bushen *Granule (BSG) P.O + Marine Injection (MI) [118]	CHB patients. Combined treatment of BSG and MI was better than Lamivudine group in one year therapeutical course.

## Action mechanisms of Chinese medicines in treating liver fibrosis

### Inhibition of viral replication

HBV and HCV infections account for most liver cirrhosis and primary liver cancer worldwide [6]. Certain Chinese medicines are anti-HBV and anti-HCV. Berberine markedly reduces viral production *in vitro *but is toxic to host cells [51]. Artemisinin and artesunate strongly inhibit viral production at concentrations that do not affect host cell viability; artesunate and lamivudine exhibit synergistic anti-HBV effects [51]. Another study shows that ascucubin inhibits HBV replication [63]. Nobiletin, the active ingredient of *Citrus unshiu *peel, has anti-HCV effects [94]. Clinical studies show that oxymatrine [28] is effective in reducing hepatitis B viral replication in patients with chronic hepatitis B. *Xiao Chaihu Tang *enhances production of interferon-gamma (IFN-γ) and antibodies against hepatitis B core and e antigen by peripheral blood mononuclear cells (PBMC) in patients with chronic hepatitis [82]. *Handan Ganle *inhibits viral DNA replication in patients with decompensated cirrhosis thereby leading to clinical improvement [102].

### Immunomodulation action

*Buzhong Yiqi Tang *and *Renshen Yangrong Tang *demonstrate immunomodulation effects [91]. In a study on porcine serum-induced liver fibrosis in rats [92], Interleukin 13 (IL-13) levels are positively correlated with hydroxyproline (Hyp) contents in the liver. *Buzhong Yiqi Tang *and *Renshen Yangrong Tang *significantly suppress the increase of hepatic Hyp, while *Xiao Chaihu Tang *does not. Short-term and long-term studies [93] show that *Renshen Yangrong Tang *is effective in liver fibrosis. Further studies find that *Renshen Yangrong Tang *inhibits HCV infection, and that Gomisin A, an active component in the formula's *Schisandra *fruit, exhibits protective effects on immunological hepatopathy [94].

### Anti-oxidation and anti-inflammation actions

*Salvia miltiorrhizae *(*Danshen*) extract [13] improves serum superoxide dismutase (SOD) activity and reduces malondialdehyde (MDA) content in both carbon tetrachloride (CCl_4_) and dimethylnitrosamine (DMN) induced hepatic fibrosis rat models. *Salvia miltiorrhizae *extract [18] increases hepatic glutathione levels and decreases peroxidation products in a dose-dependent manner. Taurine [27,28] reduces oxidative stress and prevents progression of hepatic fibrosis in CCl_4_-induced hepatic damaged rats and inhibits transformation of the hepatic stellate cell (HSC). In chronic ethanol-induced hepatotoxicity or CCl_4_-induced rat liver fibrosis, *Panax notoginseng *(*Tianqi*) extract or total saponin extracted from *Panax notoginseng *reduces the generation of MDA, scavenges free radicals, increases liver and serum SOD content and reduces the accumulation of body lipid peroxide [44-46]. *Ginkgo biloba *(*Yinxing*) extract [49,50] and berberine [54,55,60] exhibit anti-oxidation effects and suppress nuclear factor κB (NF-κB) in rats or cell culture. *Yinchenhao Tang *is used to treat liver fibrosis and portal hypertension through suppressing the activated HSC function by genipin, an absorbed form of its component, in CCl_4_-or pig-serum- induced rat liver fibrosis [72]. Lin *et al*. [68] find that the hepatoprotective effect of *Solanum nigrum *Linn extract on CCl_4_-induced liver fibrosis is achieved through blocking oxidative stress. *Xiao Chaihu Tang *[76,83,85] whose active components baicalin and baicalein function as a potent fibrosis suppressant via the inhibition of the oxidative stress in hepatocyte and HSC. *Handan Ganle *[99] is effective in protecting against liver fibrosis by inhibiting lipid peroxidation in hepatocytes and HSC *in vivo*.

### Regulation of cytokines, collagen metabolism and inhibition of HSC

The fibrogenic process is regulated by TGF-β1 and the specific blockade of TGF-β1/Smad3 signalling may therapeutically intervene in the fibrosis of various tissues [119]. Most of the Chinese medicines listed in Tables 1 and 2 exhibit *in vitro *and *in vivo *inhibitory effects on TGF-β1. Salvianolic acid B (SA-B) inhibits HSC proliferation and collagen production and decreases the cellular TGF-β1 autocrine and Mitogen-Activated Protein Kinase (MAPK) activity, which may be the anti-fibrosis mechanism of SA-B [14,17]. Paclitaxel, a compound isolated from *Taxus brevifolia*, suppresses the TGF-β1 signalling pathway between biliary epithelium cells and myofibroblasts and reduces collagen synthesis [120]. *Yinchenhao Tang *[71] regulates platelet-derived growth factor (PDGF)-BB-dependent signalling pathways of HSC in primary culture and attenuates the development of liver fibrosis induced by thioacetamide in rats. Among the components of *Yinchenhao Tang*, 3-methyl-1,6,8-trihydroxyanthraquinone (emodin) derived from *Rhei rhizoma *is the most active compound [72]. Genipin, a metabolite derived from *Yinchenhao Tang*, suppresses wound-induced cell migration and proliferation and decreases collagen type I, TGF β1 and α-smooth muscle actins (α-SMA) mRNA and protein expression [76]. Chen *et al*. [67] demonstrate that Gypenosides inhibits PDGF-induced HSCs proliferation through inhibiting the signalling pathway of PDGF-Akt-p70^S6K ^and down-regulating cyclin D1 and D3 expression. Another study shows that ganoderic acids and ganoderenic acids in *Ganoderma lucidum *(*Lingzhi*) extract significantly inhibit the proliferation of HSCs by attenuating the blockade of PDGFβR phosphorylation [66]. Chen *et al*. [88] show that 0.5 g/kg/day of *Xiao Chaihu Tang *significantly reduces the serum level of the N-terminal pro-peptide of collagen type III (PIII NP) and the mRNA expression of TGF-β1 and PDGF in a rat bile duct ligated model.

### Anti-apoptosis in hepatocyte and inducement of apoptosis in HSC

Yamamoto *et al*. [73] find that *Yinchenhao Tang *inhibits hepatocyte apoptosis induced by TGF-β1 *in vitro*. Another study [74] demonstrates that pre-treatment with *Yinchenhao Tang *markedly suppresses liver apoptosis/injury. Genipin, which is a principal ingredient of *Yinchenhao Tang*, suppresses Fas-mediated apoptosis in primary-cultured murine hepatocytes *in vitro *[73]. The resistance to Ca^2+^-induced mitochondrial permeability transition (MPT) is enhanced in liver mitochondria of genipin-treated mice [74]. These results suggest that the anti-apoptotic activity of genipin via the interference with MPT is a possible mechanism for the therapeutic effects of *Yinchenhao Tang *and that *Yinchenhao Tang *and its ingredient genipin protect hepatocyte from liver apoptosis/injury. Conversely, activated HSC plays a pivotal role in hepatic fibrosis, HSC apoptosis is involved in the mechanisms of spontaneous resolution of rat hepatic fibrosis, and the agent that induces HSC apoptosis has been shown to reduce experimental hepatic fibrosis in rats [121]. Considerable interest has been generated in uncovering the molecular events that regulate HSC apoptosis and discovering drugs that can stimulate HSC apoptosis in a selective manner. Ikeda *et al*. [75] find that *Yinchenhao Tang *induces HSC apoptosis in a time- and concentration-dependent manner as judged by the nuclear morphology, quantitation of cytoplasmic histone-associated DNA oligonucleosome fragments and caspase-3 activity. Thus, the induction of HSC apoptosis may be the mechanism whereby *Yinchenhao Tang *treats hepatic fibrosis. Tetrandrine [29] also induces apoptosis of T-HSC/Cl-6 cells and induces the activation of caspase-3 protease and subsequent proteolytic cleavage of poly (ADP-ribose) polymerase.

### Synergistic effects on liver fibrosis and carcinogenesis

Berberine derived from berberis markedly reduces viral production *in vitro *[51]. In liver damage induced by paracetamol or CCl_4_, *Berberis aristata *fruit extract and berberine, its principal ingredient, show hepato-protective action [52,53]. Berberine also exhibits antioxidative effects on tert-butyl hydroperoxide-induced oxidative damage in rat liver [54] and in the lipopolysaccharide (LPS) plus ischemia-reperfusion model [55]. Berberine abolishes acetaldehyde-induced NF-κB activity and cytokine production in a dose dependent manner, suggesting the potential role of berberine to treat alcoholic liver disease (ALD) [56]. In the rat liver fibrosis induced by multiple hepatotoxic factors (CCl_4_, ethanol and high cholesterol), the serum levels of ALT and AST and the hepatic content of MDA and Hyp are markedly decreased, while the activity of hepatic SOD is significantly increased in berberine-treated groups in a dose-dependent manner. In addition, histopathological changes, such as steatosis, necrosis and myofibroblast proliferation, are reduced and the expression of α-SMA and TGF-β1 is significantly down-regulated in the berberine-treated groups [57].

Clinically, berberine has been used in Japan to alleviate hypertyraminemia in patients with liver cirrhosis [58]. Berberine possesses anti-tumor effects in rats and mice with chemical-induced liver cancer [59] and anti-invasion in human lung cancer cell lines [60]. The mechanism may be related to its anti-inflammation effects [60,61]. The inhibitory effects of two different doses of berberine in human liver cancer HepG2 cell lines display different effects: in HepG2 cells treated with 24.0 mg/L of berberine, an increase in the sub G_0 _phase that indicates cell death is observed in cell cycle analysis with flow cytometry, however, there is no significant increase in sub G_0 _in HepG2 cells treated with 4.0 mg/L of berberine [62]. These results demonstrate that the dosage of berberine is a meaningful factor in liver diseases treatment. Composite formulae, such as *Xiao Chaihu Tang*, not only inhibit viral replication, ameliorate inflammation and enhance regeneration of hepatic cells, but also inhibit HSC proliferation, suppress intra- and extra-cellular secretion, decrease the secretion of collagen and promote its degradation and re-absorption [79-90]. Shimizu *et al*. [83] show that *Xiao Chaihu Tang *functions as a potent anti-fibrosis agent via the inhibition of oxidative stress in hepatocytes and HSCs and that its active components are baicalin and baicalein. It should be noted that baicalin and baicalein are flavonoids with chemical structures very similar to silybinin which possess anti-fibrogenic activities. Several composite formulae have been used to improve ascites induced by hepatic cirrhosis in chronic hepatitis B (CHB) or chronic hepatitis C (CHC) patients. We demonstrate that *Buqi Jianzhong Tang *and *Fangji Tang *increase Na^+ ^excretion and urine volume and reduce GOT and GPT in rats with CCl_4_- induced liver damage [89,98]. Most of the Chinese medicines in Tables 1 and 2 reduce serum enzymes, i.e. aspartate transaminase (AST) and alanine transaminase (ALT). A study with multivariate analysis demonstrates that the mode of therapy and ALT levels are significant factors affecting HCC development [26]. Glycyrrhizin administered as Stronger Neo Minophagen C (SNMC) and *Xiao Chaihu Tang *exhibit this effect [24-26,90] in long-term clinical trials. Considered to possess anti-carcinogenic properties, *Xiao Chaihu Tang *inhibits chemical hepatocarcinogenesis in animals, acts as a biological response modifier and suppresses the proliferation of hepatoma cells by inducing apoptosis and arresting the cell cycle. Among the active components of *Xiao Chaihu Tang*, baicalin, baicalein and saikosaponin have the ability to inhibit cell proliferation [90].

## Efficiency and safety of Chinese medicines in treating liver fibrosis

### Efficacy

Some anti-fibrosis Chinese medicines, such as Salvianolic acid B (SA-B), tetrandrine and oxymatrine, are clinically effective. SA-B reverses liver fibrosis in chronic hepatitis B patients. SA-B reduces the serum HA content and decreases the overall serum fibrosis markers better than IFN-γ [14]. A multi-centre, randomized, double-blind, placebo-controlled clinical trial shows that oxymatrine effectively reduces the DNA replication of HBV [34,35] and the therapeutic effect is even stronger when used together with *Xiao Chaihu Tang *[110]. A double-blind, randomized, placebo-controlled phases I/II trial of intravenous glycyrrhizin for the treatment of chronic hepatitis C shows that glycyrrhizin lowers serum ALT and that the treatment has no effect on the RNA levels of HCV [23]. Long-term clinical trials in Japan and the Netherlands demonstrate that interferon non-responder patients with chronic hepatitis C and fibrosis stage 3 or 4 have a reduced incidence rate of HCC after glycyrrhizin therapy normalizes ALT levels [24,25].

In China and Japan, many composite formulae are used to treat liver fibrosis and cirrhosis (Table 2) and the pharmacological effects and mechanisms have been demonstrated [69-94]. Experimental and clinical studies show that *Handan Ganle *is effective [99-102]. *Fuzheng Huayu*, another modern formula, has also been intensively studied [104-107]. The results suggest that *Fuzheng Huayu*'s anti-fibrosis effects may be associated with the inhibition of liver collagen production [104]. Further study reveals that the conditioned medium from activated HSC stimulates the quiescent HSC proliferation and type I collagen secretion and that the drug serum inhibits this stimulating action and vascular endothelial growth factor (VEGF) secretion from the activated HSC. *Fuzheng Huayu *acts effectively against the autocrine activation pathway of HSC [105].

A recent study demonstrates the action of *Fuzheng Huayu *against HSC activation via the fibronectin/integrin-5β1 signalling pathway [107]. Another study shows that *Fuzheng Huayu *alleviates liver fibrosis without any adverse events [106]. A systematic review analyzes the efficacy and safety of *Fuzheng Huayu *in treatment of CHB fibrosis [108] based on clinical trials with placebo and/or random control (other positive Chinese medicines and conventional drugs). Seven studies on *Fuzheng Huayu *in the treatment of CHB fibrosis (total 590 cases) are included in the systematic review. This systematic review concludes that *Fuzheng Huayu *has significant improvement of serum fibrosis index and pathology of liver biopsy (class S in fibrosis) without observable adverse events, although some included studies are of low quality and are small randomized clinical trials.

The combined therapy with ursodeoxycholic acid and glycyrrhizin is safe and effective in improving liver-specific enzyme abnormalities, and may be an alternative to interferon in chronic hepatitis C viral infection, especially for interferon-resistant or unstable patients [110]. The antiviral efficacy of *Bushen *granule (BSG) coupled with marine injection (MI) to treat chronic hepatitis B was more effective than lamivudine treatment [118]. Other reports of therapeutic value gained through combining conventional and Chinese medicines can be found in Table 3[112-117].

### Safety

There have been reports on adverse events and hepatotoxicity caused by herbal medicines [122]. *Xiao Chaihu Tang*, used alone or in combination with interferon, may induce acute interstitial pneumonia in patients with chronic active hepatitis [113,114]. Glycyrrhizin injection may induce fatal biliary cirrhosis [123]. A one-year study demonstrates that Chinese medicines caused hepatotoxicity in patients with chronic hepatitis B [124]. Some of hepatic veno-occlusive diseases have been ascribed to toxicity of herbs; however, the toxic compounds remain to be determined. Hepatic veno-occlusive disease may result from pyrrolizidine alkaloids which are found in numerous plants worldwide. Systematic toxicological knowledge of Chinese medicines is available [125].

Adverse events in the cases of herbal toxicity are in fact very complex. The fatal biliary cirrhosis case [123] was a 50-year-old woman suffering from a diffuse skin rash, high fever and jaundice immediately after a second injection of glutathione and stronger neo-minophagen C, which contains glycyrrhizin. It is difficult to determine the cause of the adverse events to be indeed glycyrrhizin (which is extracted from *Glycyrrhiza uralensis*) for the following reasons: (1) no literature has shown the hepatotoxicity of glycyrrhizin until now; (2) stronger neo-minophagen C includes 0.1% cysteine and 2.0% glycine in physiological saline solution as well as 0.2% glycyrrhizin, and is also combined with glutathione; and (3) the clinical indication of glycyrrhizin was clear enough (glycyrrhizin is only used in chronic liver hepatitis without bile duct obstruction, which is *Yinchenhao Tang*'s indication in Chinese medicine clinical practice), and glycyrrhizin has no anti-fibrotic effect in rats with fibrosis induced by bile duct ligation and scission [65].

### Evidence against Chinese medicines

While ample evidence supports Chinese medicines in treating liver fibrosis, some recent reviews on clinical trials did not find significant effects. Levy *et al*. [126] review the use of silymarin, glycyrrhizin, *Xiao Chaihu Tang*, *Phyllanthus amarus*, *Picrorrhiza kurroa*, Compound 861, CH-100 and LIV.52 used to treat chronic liver diseases. Dhiman *et al*. [127] review *Phyllanthus*, *Silybum marianum *(milk thistle), glycyrrhizin and LIV.52 used to treat liver diseases. However, neither review recommends the use of herbal medicines to treat chronic liver diseases.

SA-B, Glycyrrhizin, *Xiao Chaihu Tang *and *Yinchenhao Tang *are used to treat chronic liver diseases in China and Japan. The major active herb is coptis, of which berberine is the major active component [128]. According to Chinese medicine theory, we use coptis to treat various liver diseases and cancer in Hong Kong [129]. We also propose to replace bear bile with coptis in Chinese medicine practice [130].

**Figure 1:**
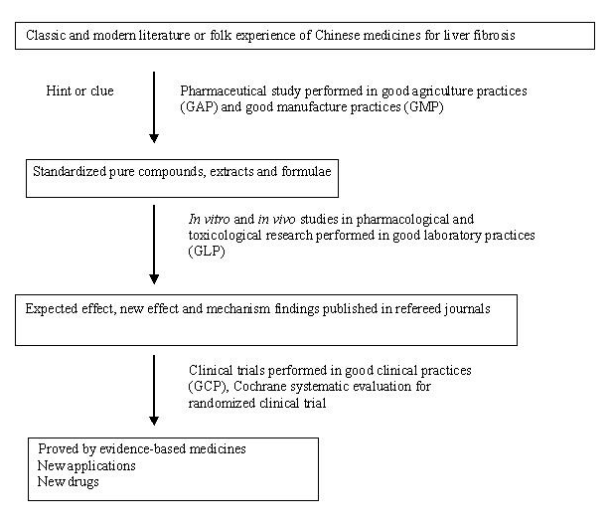
**Research chart of Chinese medicines for liver fibrosis**. The re-evaluation involved in pharmaceutical and medical research including herb quality control, mechanism study and clinical trial will be carried out on standardized international platforms.

Further studies on pharmacological actions and clinical efficacies of the anti-fibrosis effects of Chinese medicines are warranted. Systematic reviews to evaluate clinical studies on the efficacy and safety of Chinese medicines are also necessary. An exemplifying strategy for these studies is demonstrated in Figure 1.

## Conclusion

Evidence indicates that some Chinese medicines are clinically effective in treating liver fibrosis. Strict quality control of Chinese medicines is critical [131] for pharmacological, clinical and in-depth mechanism studies [132]. Experiments and clinical trials should be carried out on the platforms that conform to international standards [133].

## Abbreviations

ECM: extracellular matrix; HSC: hepatic stellate cell; CAM: complementary and alternative medicine; SA-B: salvianolic acid B; HBV: hepatitis B virus; HCV: hepatitis C virus; CHB: chronic hepatitis B; CHC: chronic hepatitis C; AST (= GOT): aspartate aminotransferase; ALT (= GPT): alanine aminotranferease; TGF-β1: transforming growth factor beta1; Smad3: SMAD family member 3; Smad7: SMAD family member 7; smurf2: Smad ubiquitination regulatory factor 2; TIMP: tissue inhibitors of metalloproteases; MMP: matrix metalloproteinase; MAPK: mitogen-activated protein kinase; NF-κB: nuclear factor-κB; PDGF: platelet-derived growth factor; PPARgamma: proliferator-activated receptor gamma; SOD: superoxide dismutase; Hyp: hydroxyproline; HA: hyaluronic acid; α-SMA: α-smooth muscle actins; IFN-γ: interferon-gamma; IFN-α: interferon-alfa; LN: laminin; PCIII: type III procollagen; CIV: type IV collagen; Tbil: total bilirubin; TNF-α: tumor necrosis factor alfa; PIIINP: the N-terminal pro-peptide of collagen type III; MPT: mitochondrial permeability transition; Alb: albumin; BCAA: branched chain amino acid; AAA: aromatic amino acid; FN/integrin: fibronectin (FN)-integrin-5β1 complex.

## Competing interests

*Fuzhen Huayu *is a herbal product developed by PL's institution at the Shanghai University of Traditional Chinese Medicine. The authors declare that they have no competing interests for other Chinese medicines discussed in the present study.

## Authors' contributions

YBF and YT conceived the study, interpreted the data and revised the manuscript. YBF retrieved and analyzed the data and drafted the manuscript. KFC and NW retrieved the data from Chinese journals and translated them into English. PL and TN supervised some of the experiments. All authors read and approved the final version of the manuscript.

## References

[B1] Yuen MF Lai CL Natural history of chronic hepatitis B virus infection J Gastroenterol Hepatol 2000 15 Suppl E20 4 10921377 10.1046/j.1440-1746.2000.02123.x

[B2] Seeff LB Hoofnagle JH The national institutes of health consensus development conference management of hepatitis C 2002 Clin Liver Dis 2003 7 261 87 12691470 10.1016/S1089-3261(02)00078-8

[B3] Koike K Antiviral treatment of hepatitis C: present status and future prospects J Infect Chemother 2006 12 227 32 17109084 10.1007/s10156-006-0460-0

[B4] Tsukamoto H Lu SC Current concepts in the pathogenesis of alcoholic liver injury FASEB J 2001 15 1335 49 11387231 10.1096/fj.00-0650rev

[B5] Heidelbaugh JJ Bruderly M Cirrhosis and chronic liver failure: part I. Diagnosis and evaluation Am Fam Physician 2006 74 756 62 16970019

[B6] Perz JF Armstrong GL Farrington LA Hutin YJ Bell BP The contributions of hepatitis B virus and hepatitis C virus infections to cirrhosis and primary liver cancer worldwide J Hepatol 2006 45 529 38 16879891 10.1016/j.jhep.2006.05.013

[B7] Wells RG The role of matrix stiffness in hepatic stellate cell activation and liver fibrosis J Clin Gastroenterol 2005 39 158 61 10.1097/01.mcg.0000155516.02468.0f 15758652

[B8] Friedman SL Liver fibrosis–from bench to bedside J Hepatol 2003 38 Suppl 1 38 53 10.1016/S0168-8278(02)00429-4 12591185

[B9] Friedman SL Reversibility of hepatic fibrosis and cirrhosis–is it all hype? Nat Clin Pract Gastroenterol Hepatol 2007 4 5 236 7 17476207 10.1038/ncpgasthep0813

[B10] Schuppan D Jia JD Brinkhaus B Hahn EG Herbal products for liver diseases: a therapeutic challenge for the new millennium Hepatolog 1999 30 4 1099 104 10.1002/hep.510300437 10498665

[B11] Shimizu I Antifibrogenic therapies in chronic HCV infection Infect Disord Drug Targets 2001 1 2 227 40 10.2174/1568005014606053 12455417

[B12] Seeff LB Lindsay KL Bacon BR Kresina TF Hoofnagle JH Complementary and alternative medicine in chronic liver disease Hepatology 2001 34 3 595 603 11526548 10.1053/jhep.2001.27445

[B13] Xie HM Hu YY Gu HT Nagano Y Ji G Liu P Study of Salviae miltiorrhizae on liver fibrosis in rats induced by CCl4 and DMN Zhongxiyi Jiehe Ganbing Zazh 1999 9 16 8

[B14] Liu P Hu YY Liu C Zhu DY Xue HM Xu ZQ Xu LM Liu CH Gu HT Zhang ZQ Clinical observation of salvianolic acid B in treatment of liver fibrosis in chronic hepatitis B World J Gastroenterol 2002 8 679 85 12174378 10.3748/wjg.v8.i4.679 PMC4656320

[B15] Lee TY Wang GJ Chiu JH Lin HC Long-term administration of *Salvia miltiorrhiza *ameliorates carbon tetrachloride-induced hepatic fibrosis in rats J Pharm Pharmacol 2003 55 1561 8 14713368 10.1211/0022357022098

[B16] Wang H Chen XP Qiu FZ Salviae miltiorrhizae ameliorates cirrhosis and portal hypertension by inhibiting nitric oxide in cirrhotic rats Hepatobiliary Pancreat Dis Int 2003 2 391 6 14599946

[B17] Zhao JF Liu CH Hu YY Xu LM Liu P Liu C Effect of salvianolic acid B on Smad3 expression in hepatic stellate cells Hepatobiliary Pancreat Dis Int 2004 3 102 5 14969849

[B18] Lee TY Chang HH Wang GJ Chiu JH Yang YY Lin HC Water-soluble extract of *Salvia miltiorrhiza *ameliorates carbon tetrachloride-mediated hepatic apoptosis in rats J Pharm Pharmacol 2006 58 659 65 16640835 10.1211/jpp.58.5.0011

[B19] Yamamura Y Kotaki H Tanaka N Aikawa T Sawada Y Iga T The pharmacokinetics of glycyrrhizin and its restorative effect on hepatic function in patients with chronic hepatitis and in chronically carbon-tetrachloride-intoxicated rats Biopharm Drug Dispos 1997 18 717 25 9373728 10.1002/(SICI)1099-081X(199711)18:8<717::AID-BDD54>3.0.CO;2-U

[B20] Wang JY Guo JS Li H Liu SL Zern MA Inhibitory effect of glycyrrhizin on NF-kappaB binding activity in CCl4- plus ethanol-induced liver cirrhosis in rats Liver 1998 18 180 5 9716228 10.1111/j.1600-0676.1998.tb00147.x

[B21] Iino S Tango T Matsushima T Toda G Miyake K Hino K Kumada H Yasuda K Kuroki T Hirayama C Suzuki H Therapeutic effects of stronger neo-minophagen C at different doses on chronic hepatitis and liver cirrhosis Hepatol Res 2001 19 31 40 11137478 10.1016/S1386-6346(00)00079-6

[B22] Cai Y Shen SZ Wang JY Effects of glycyrrhizin on genes expression during the process of liver fibrosis Zhonghua Yixue Zazhi 2003 83 1122 5 12921627

[B23] Rossum Van TGJ Vulto AG Hop WC Brouwer JT Niesters HG Schalm SW Intravenous glycyrrhizin for the treatment of chronic hepatitis C: a double blind, randomised, placebo controlled phase I/II trial J Gastroenterol Hepatol 1999 14 1093 9 10574137 10.1046/j.1440-1746.1999.02008.x

[B24] Veldt BJ Hansen BE Ikeda K Verhey E Suzuki H Schalm SW Long-term clinical outcome and effect of glycyrrhizin in 1093 chronic hepatitis C patients with non-response or relapse to interferon Scand J Gastroenterol 2006 41 1087 94 16938723 10.1080/00365520600641365

[B25] Ikeda K Arase Y Kobayashi M Saitoh S Someya T Hosaka T Sezaki H Akuta N Suzuki Y Suzuki F Kumada H A long-term glycyrrhizin injection therapy reduces hepatocellular carcinogenesis rate in patients with interferon-resistant active chronic hepatitis C: a cohort study of 1249 patients Dig Dis Sci 2006 51 603 9 16614974 10.1007/s10620-006-3177-0

[B26] Rino Y Tarao K Morinaga S Ohkawa S Miyakawa K Hirokawa S Masaki T Tarao N Yukawa N Saeki H Takanashi Y Imada T Reduction therapy of alanine aminotransferase levels prevent HCC development in patients with HCV-associated cirrhosis Anticancer Res 2006 26 3B 2221 6 16821591

[B27] Wang ZR Chen XM Li DG Tetrandrine inhibits expressions of c-fos and c-jun mRNA in fibrosis liver of rats Shanghai Yixue 2003 26 332 4

[B28] Wang H Chen XP Qiu FZ Tetrandrine increased hepatic expression of nitric oxide synthase type II in cirrhotic rats World J Gastroenterol 2004 10 1923 7 15222038 10.3748/wjg.v10.i13.1923 PMC4572232

[B29] Zhao YZ Kim JY Park EJ Lee SH Woo SW Ko G Sohn DH Tetrandrine induces apoptosis in hepatic stellate cells Phytother Res 2004 18 306 9 15162366 10.1002/ptr.1435

[B30] Chen YW Li DG Wu JX Chen YW Lu HM Tetrandrine inhibits activation of rat hepatic stellate cells stimulated by transforming growth factor-beta in vitro via up-regulation of Smad 7 J Ethnopharmacol 2005 100 299 305 15905052 10.1016/j.jep.2005.03.027

[B31] Hsu YC Chiu YT Lee CY Wu CF Huang YT Anti-fibrotic effects of tetrandrine on bile-duct ligated rats Can J Physiol Pharmacol 2006 84 967 76 17218962 10.1139/Y06-050

[B32] Hsu YC Chiu YT Cheng CC Wu CF Lin YL Huang YT Antifibrotic effects of tetrandrine on hepatic stellate cells and rats with liver fibrosis J Gastroenterol Hepatol 2007 22 99 111 17201889 10.1111/j.1440-1746.2006.04361.x

[B33] Zhang JP Zhang M Zhou JP Liu FT Zhou B Xie WF Guo C Antifibrotic effects of matrine on in vitro and in vivo models of liver fibrosis in rats Acta Pharmacol Sin 2001 22 183 6 11741525

[B34] Chen YX Mao BY Jiang JH Relationship between serum load of HBV-DNA and therapeutic effect of oxymatrine in patients with chronic hepatitis B Zhongguo Zhongxiyi Jiehe Zazhi 2002 22 335 6 12584828

[B35] Mao YM Zeng MD Lu LG Capsule oxymatrine in treatment of hepatic fibrosis due to chronic viral hepatitis: a randomized, double blind, placebo-controlled, multicenter clinical study World J Gastroenterol 2004 10 3269 73 15484298 10.3748/wjg.v10.i22.3269 PMC4572293

[B36] Kato J Ido A Hasuike S Uto H Hori T Hayashi K Murakami S Terano A Tsubouchi H Transforming growth factor-beta-induced stimulation of formation of collagen fiber network and anti-fibrotic effect of taurine in an in vitro model of hepatic fibrosis Hepatol Res 2004 30 34 41 15341772 10.1016/j.hepres.2004.04.006

[B37] Miyazaki T Karube M Matsuzaki Y Ikegami T Doy M Tanaka N Bouscarel B Taurine inhibits oxidative damage and prevents fibrosis in carbon tetrachloride-induced hepatic fibrosis J Hepatol 2005 43 117 25 15893842 10.1016/j.jhep.2005.01.033

[B38] Chen ZZ Wang H Inhibitory Effects of tetramethylpyrazine on experimental hepatic fibrosis in rats Zhong Xiyi Jiehe Ganbing Zazhi 1997 7 156 8

[B39] Tan LX Li XS Liu ZQ Liu LY Effects of combination therapy of rehin and tetramethylpyrazine on experimental hepatic fibrosis induced by tetrachloride Zhonghua Ganzangbing Zazhi 2004 12 692 3 15623384

[B40] Zhan Y Li D Wei H Wang Z Huang X Xu Q Lu H Emodin on hepatic fibrosis in rats Chin Med J (Engl) 2000 113 599 601 11776026

[B41] Zhan YT Liu B Li DG Bi CS Mechanism of emodin for anti-fibrosis of liver Zhonghua Ganzangbing Zazhi 2004 12 245 6 15099485

[B42] Yang W Chen H Jiang Y Inhibitive effect of curcumin and amiloride on the fibrosis of rat hepatic stellate cells induced by oxidative stress Zhongyaocai 2003 26 795 8 14989060

[B43] Zheng S Chen A Activation of PPARgamma is required for curcumin to induce apoptosis and to inhibit the expression of extracellular matrix genes in hepatic stellate cells in vitro Biochem J 2004 384 149 57 15320868 10.1042/BJ20040928 PMC1134098

[B44] Lin CF Wong KL Wu RS Huang TC Liu CF Protection by hot water extract of Panax notoginseng on chronic ethanol-induced hepatotoxicity Phytother Res 2003 17 1119 22 14595601 10.1002/ptr.1329

[B45] Shii XF Liu Q Liu L Xu M Effect of total saponin of Panax Notoginseng on liver fibrosis in rats Zhongyao Yaoli yu Linchuang 2004 20 12 4

[B46] Park WH Lee SK Kim CH A Korean herbal medicine, Panax notoginseng, prevents liver fibrosis and hepatic microvascular dysfunction in rats Life Sci 2005 76 15 1675 90 15698847 10.1016/j.lfs.2004.07.030

[B47] Gong HY Wang KQ Tang SG Effects of cordyceps sinensis on T lymphocyte subsets and hepatofibrosis in patients with chronic hepatitis B Hunan Yike Daxue Xuebao 2000 25 248 50 12212155

[B48] Liu YK Shen W Inhibitive effect of cordyceps sinensis on experimental hepatic fibrosis and its possible mechanism World J Gastroenterol 2003 9 529 33 12632512 10.3748/wjg.v9.i3.529 PMC4621576

[B49] Ding J Yu J Wang C Hu W Li D Luo Y Luo H Yu H Ginkgo biloba extract alleviates liver fibrosis induced by CCl4 in rats Liver International 2005 25 1224 32 16343076 10.1111/j.1478-3231.2005.01169.x

[B50] Liu SQ Yu JP Chen HL Luo HS Chen SM Yu HG Therapeutic effects and molecular mechanisms of Ginkgo biloba extract on liver fibrosis in rats Am J Chin Med 2006 34 99 114 16437743 10.1142/S0192415X06003679

[B51] Romero MR Efferth T Serrano MA Castano B Macias RI Briz O Marin JJ Effect of artemisinin/artesunate as inhibitors of hepatitis B virus production in an "in vitro" replicative system Antiviral Res 2005 68 75 83 16122816 10.1016/j.antiviral.2005.07.005

[B52] Gilani AH Janbaz KH Preventive and curative effects of berberis aristata fruit extract on paracetamol- and CCl4-induced hepatotoxicity Phytotherapy Res 1995 9 489 94 10.1002/ptr.2650090705

[B53] Janbaz KH Gilani AH Studies on preventive and curative effects of berberine on chemical-induced hepatotoxicity in rodents Fitoterapia 2000 71 25 33 11449466 10.1016/S0367-326X(99)00098-2

[B54] Hwang JM Wang CJ Chou FP Tseng TH Hsieh YS Lin WL Chu CY Inhibitory effect of berberine on tert-butyl hydroperoxide-induced oxidative damage in rat liver Arch Toxicol 2002 76 664 70 12415430 10.1007/s00204-002-0351-9

[B55] Yokozawa T Ishida A Kashiwada Y Cho Ej Kim Hy Ikeshiro Y Coptidis Rhizoma: protective effects against peroxynitrite-induced oxidative damage and elucidation of its active components J Pharm Pharmacol 2004 56 547 56 15099450 10.1211/0022357023024

[B56] Hsiang CY Wu SL Cheng SE Ho TY Acetaldehyde-induced interleukin-1beta and tumor necrosis factor-alpha production is inhibited by berberine through nuclear factor-kappaB signaling pathway in HepG2 cells J Biomed Sci 2005 12 791 801 16132116 10.1007/s11373-005-9003-4

[B57] Zhang BJ Xu D Guo Y Ping J Chen LB Wang H Protection by and anti-oxidant mechanism of berberine against rat liver fibrosis induced by multiple hepatotoxic factors Clin Exp Pharmacol Physiol 2008 35 3 303 9 17973934 10.1111/j.1440-1681.2007.04819.x

[B58] Watanabe A Obata T Nagashima H Berberine therapy of hypertyraminemia in patients with liver cirrhosis Acta Med Okayama 1982 36 4 277 81 7136858 10.18926/AMO/30659

[B59] Anis KV Rajeshkumar NV Kuttan R Inhibition of chemical carcinogenesis by berberine in rats and mice J Pharm Pharmacol 2001 53 763 8 11370717 10.1211/0022357011775901

[B60] Peng PL Hsieh YS Wang CJ Hsu JL Chou FP Inhibitory effect of berberine on the invasion of human lung cancer cells via decreased productions of urokinase-plasminogen activator and matrix metalloproteinase-2 Toxicol Appl Pharmacol 2006 214 8 15 16387334 10.1016/j.taap.2005.11.010

[B61] Kuo CL Chi CW Liu TY The anti-inflammatory potential of berberine in vitro and in vivo Cancer Lett 2004 203 127 37 14732220 10.1016/j.canlet.2003.09.002

[B62] Tan Yl Goh D Ong ES Investigation of differentially expressed proteins due to the inhibitory effects of berberine in human liver cancer cell line HepG2 Mol Biosyst 2006 2 250 8 16880943 10.1039/b517116d

[B63] Chang IM Liver-protective activities of aucubin derived from traditional oriental medicine Res Commun Mol Pathol Pharmacol 1998 102 189 204 10100510

[B64] Park KS Chang IM Anti-inflammatory activity of aucubin by inhibition of tumor necrosis factor-alpha production in RAW 264.7 cells Planta Med 2004 70 778 9 15326552 10.1055/s-2004-827211

[B65] Park EJ Ko G Kim J Sohn DH Antifibrotic effects of a polysaccharide extracted from Ganoderma lucidum, glycyrrhizin, and pentoxifylline in rats with cirrhosis induced by biliary obstruction Biol Pharm Bull 1997 20 417 20 9145221 10.1248/bpb.20.417

[B66] Wang GJ Huang YJ Chen DH Lin YL Ganoderma lucidum extract attenuates the proliferation of hepatic stellate cells by blocking the PDGF receptor Phytother Res 2008 23 833 9 10.1002/ptr.2687 19107744

[B67] Chen MH Chen SH Wang QF Chen JC Chang DC Hsu SL Chen CH Sheue CR Liu YW The molecular mechanism of gypenosides-induced G1 growth arrest of rat hepatic stellate cells J Ethnopharmacol 2008 117 309 17 18372131 10.1016/j.jep.2008.02.009

[B68] Lin HM Tseng HC Wang CJ Lin JJ Lo CW Chou FP Hepatoprotective effects of Solanum nigrum Linn extract against CCl_4_-induced oxidative damage in rats Chem Biol Interact 2008 171 283 93 18045581 10.1016/j.cbi.2007.08.008

[B69] Kobayashi H Horikoshi K Yamataka A Lane GJ Yamamoto M Miyano T Beneficial effect of a traditional herbal medicine (inchin-ko-to) in postoperative biliary atresia patients Pediatr Surg Int 2001 17 386 9 11527172 10.1007/s003830000561

[B70] Sakaida I Tsuchiya M Kawaguchi K Kimura T Terai S Okita K Herbal medicine Inchin-ko-to (TJ-135) prevents liver fibrosis and enzyme-altered lesions in rat liver cirrhosis induced by a choline-deficient L-amino acid-defined diet J Hepatol 2003 38 762 9 12763369 10.1016/S0168-8278(03)00094-1

[B71] Imanishi Y Maeda N Otogawa K Seki S Matsui H Kawada N Arakawa T Herb medicine Inchin-ko-to (TJ-135) regulates PDGF-BB-dependent signaling pathways of hepatic stellate cells in primary culture and attenuates development of liver fibrosis induced by thioacetamide administration in rats J Hepatol 2004 41 242 50 15288473 10.1016/j.jhep.2004.04.005

[B72] Inao M Mochida S Matsui A Eguchi Y Yulutuz Y Wang Y Naiki K Kakinuma T Fujimori K Nagoshi S Fujiwara K Japanese herbal medicine Inchin-ko-to as a therapeutic drug for liver fibrosis J Hepatol 2004 41 584 91 15464238 10.1016/j.jhep.2004.06.033

[B73] Yamamoto M Ogawa K Morita M Fukuda K Komatsu Y The herbal medicine Inchin-ko-to inhibits liver cell apoptosis induced by transforming growth factor beta 1 Hepatology 1996 23 552 9 8617437 10.1053/jhep.1996.v23.pm0008617437

[B74] Yamamoto M Miura N Ohtake N Amagaya S Ishige A Sasaki H Komatsu Y Fukuda K Ito T Terasawa K Genipin, a metabolite derived from the herbal medicine Inchin-ko-to, and suppression of Fas-induced lethal liver apoptosis in mice Gastroenterology 2000 118 380 9 10648466 10.1016/S0016-5085(00)70220-4

[B75] Ikeda H Nagashima K Yanase M Tomiya T Arai M Inoue Y Tejima K Nishikawa T Watanabe N Kitamura K Isono T Yahagi N Noiri E Inao M Mochida S Kume Y Yatomi Y Nakahara K Omata M Fujiwara K The herbal medicine inchin-ko-to (TJ-135) induces apoptosis in cultured rat hepatic stellate cells Life Sci 2006 78 2226 33 16280138 10.1016/j.lfs.2005.09.024

[B76] Kitano A Saika S Yamanaka O Ikeda K Reinach PS Nakajima Y Okada Y Shirai K Ohnishi Y Genipin suppresses subconjunctival fibroblast migration, proliferation and myofibroblast transdifferentiation Ophthalmic Res 2006 38 6 355 60 17047408 10.1159/000096231

[B77] Lee TY Chang HH Chen JH Hsueh ML Kuo JJ Herb medicine Yin-Chen-Hao-Tang ameliorates hepatic fibrosis in bile duct ligation rats J Ethnopharmacol 2007 109 318 24 16989967 10.1016/j.jep.2006.07.042

[B78] Lee TY Chang HH Kuo JJ Shen JJ Changes of hepatic proteome in bile duct ligated rats with hepatic fibrosis following treatment with Yin-Chen-Hao-Tang Int J Mol Med 2009 23 477 84 19288023 10.3892/ijmm_00000154

[B79] Miyamura M Ono M Kyotani S Nishioka Y Effects of sho-saiko-to extract on fibrosis and regeneration of the liver in rats J Pharm Pharmacol 1998 50 97 105 9504440 10.1111/j.2042-7158.1998.tb03311.x

[B80] Sakaida I Matsumura Y Akiyama S Hayashi K Ishige A Okita K Herbal medicine Sho-saiko-to (TJ-9) prevents liver fibrosis and enzyme-altered lesions in rat liver cirrhosis induced by a choline-deficient L-amino acid-defined diet J Hepatol 1998 28 298 306 9514543 10.1016/0168-8278(88)80017-5

[B81] Kayano K Sakaida I Uchida K Okita K Inhibitory effects of the herbal medicine Sho-saiko-to (TJ-9) on cell proliferation and procollagen gene expressions in cultured rat hepatic stellate cells J Hepatol 1998 29 642 9 9824275 10.1016/S0168-8278(98)80161-X

[B82] Yamashiki M Nishimura A Huang XX Nobori T Sakaguchi S Suzuki H Effects of the Japanese herbal medicine "Sho-saiko-to" (TJ-9) on interleukin-12 production in patients with HCV-positive liver cirrhosis Dev Immunol 1999 7 17 22 10636475 10.1155/1999/62564 PMC2276037

[B83] Shimizu I Ma YR Mizobuchi Y Liu F Miura T Nakai Y Yasuda M Shiba M Horie T Amagaya S Kawada N Hori H Ito S Effects of Sho-saiko-to, a Japanese herbal medicine, on hepatic fibrosis in rats Hepatology 1999 29 1 149 60 9862861 10.1002/hep.510290108

[B84] Ono M Miyamura M Kyotani S Saibara T Ohnishi S Nishioka Y Effects of Sho-saiko-to extract on liver fibrosis in relation to the changes in hydroxyproline and retinoid levels of the liver in rats J Pharm Pharmacol 1999 51 1079 84 10528993 10.1211/0022357991773429

[B85] Kusunose M Qiu B Cui T Hamada A Yoshioka S Ono M Miyamura M Kyotani S Nishioka Y Effect of Sho-saiko-to extract on hepatic inflammation and fibrosis in dimethylnitrosamine induced liver injury rats Biol Pharm Bull 2002 25 11 1417 21 12419951 10.1248/bpb.25.1417

[B86] Kitade Y Watanabe S Masaki T Nishioka M Nishino H Inhibition of liver fibrosis in LEC rats by a carotenoid, lycopene, or a herbal medicine, Sho-saiko-to Hepatol Res 2002 22 196 205 11882416 10.1016/S1386-6346(01)00132-2

[B87] Sakaida I Hironaka K Kimura T Terai S Yamasaki T Okita K Herbal medicine Sho-saiko-to (TJ-9) increases expression matrix metalloproteinases (MMPs) with reduced expression of tissue inhibitor of metalloproteinases (TIMPs) in rat stellate cell Life Sci 2004 74 2251 63 14987950 10.1016/j.lfs.2003.09.059

[B88] Chen MH Chen JC Tsai CC Wang WC Chang DC Tu DG Hsieh HY The role of TGF-beta 1 and cytokines in the modulation of liver fibrosis by Sho-saiko-to in rat's bile duct ligated model J Ethnopharmacol 2005 97 1 7 13 15652268 10.1016/j.jep.2004.09.040

[B89] Kakumu S Yoshioka K Wakita T Ishikawa T Effect of TJ-9 Sho-saiko-to (Kampo medicine) on interferon gamma and antibody production specific for hepatitis B virus antigen in patients with type B chronic hepatitis Int Immunopharmacol 1991 13 141 6 10.1016/0192-0561(91)90091-K 1906436

[B90] Shimizu I Sho-saiko-to: Japanese herbal medicine for protection against hepatic fibrosis and carcinoma J Gastroenterol Hepatol 2000 15 Suppl D84 90 10759225 10.1046/j.1440-1746.2000.02138.x

[B91] Abe S Ishibshi H Tansho S Hanazawa R Komatsu Y Yamaguchi H Protective effect of oral administration of several traditional Kampo-medicines on lethal Candida infection in immunosuppressed mice Nippon Ishinkin Gakkai Zasshi 2000 41 115 9 10777823 10.3314/jjmm.41.115

[B92] Ochi T Kawakita T Nomoto K Effects of Hochu-ekki-to and Ninjin-youei-to, traditional Japanese medicines, on porcine serum-induced liver fibrosis in rats Immunopharmacol Immunotoxicol 2004 26 285 98 15209364 10.1081/IPH-120037726

[B93] Cyong JC Ki SM Iijima K Kobayashi T Furuya M Clinical and pharmacological studies on liver diseases treated with Kampo herbal medicine Am J Chin Med 2000 28 351 60 11154048 10.1142/S0192415X00000416

[B94] Suzuki M Sasaki K Yoshizaki F Oguchi K Fujisawa M Cyong JC Anti-hepatitis C virus effect of citrus unshiu peel and its active ingredient nobiletin Am J Chin Med 2005 33 87 94 15844836 10.1142/S0192415X05002680

[B95] Song SL Gong ZJ Zhang QR Therapeutic effect and mechanism of traditional Chinese compound decoction of Radix Curcumae, Rhzoma Sparganii, Rhizoma Zedoariae on fibrotic liver in rats Zhong Caoyao 2004 35 293 6

[B96] Lu ZL Li JC Liu JD Experimental study of effects of TCM formulas on regulation of peritoneal lymphatic stomata and urine sodium in liver fibrosis mouse model Zhongguo Zhongyiyao Xinxi Zazhi 2000 7 25 6

[B97] Feng Y Nagamatu T Suzuki Y Kawata T Koike T The diuretic effects of Wakan-yaku prescription on normal rats and various pathological models Wakan Iyakugaku Zasshi 1996 13 484 485

[B98] Feng Y Nagamatu T Suzuki Y Kawata T Feng YG Kobayashi S Koike T Pharmacological studies of diuretic Wakan-yaku formulations: its application and evaluation of Pharmacological screening Wakan Iyakugaku Zasshi 2000 17 122 30

[B99] Li CX Li L Lou J Yang WX Lei TW Li YH Liu J Cheng ML Huang LH The protective effects of traditional Chinese medicine prescription, han-dan-gan-le, on CCl4-induced liver fibrosis in rats Am J Chin Med 1998 26 325 32 9862020 10.1142/S0192415X98000361

[B100] Li C Luo J Li L Cheng M Huang N Liu J Waalkes MP The collagenolytic effects of the traditional Chinese medicine preparation, Han-Dan-Gan-Le, contribute to reversal of chemical-induced liver fibrosis in rats Life Sci 2003 72 1563 71 12551745 10.1016/S0024-3205(02)02448-7

[B101] Yang Q Xie RJ Geng XX Luo XH Han B Cheng ML Effect of Danshao Huaxian capsule on expression of matrix metalloproteinase-1 and tissue inhibitor of metalloproteinase-1 in fibrotic liver of rats World J Gastroenterol 2005 11 4953 6 16124044 10.3748/wjg.v11.i32.4953 PMC4321908

[B102] Cheng ML Lu T Yao YM Geng XX Danshao huaxian capsule in treatment of decompensated cirrhosis resulting from chronic hepatitis B Hepatobiliary Pancreat Dis Int 2006 5 48 51 16481282

[B103] Wang LC Zhao LS Tang H Experimental study of liver fibrosis reversal effect of warming-yang compound formula ganzhifu Zhongguo Zhongxiyi Jiehe Zazhi 2006 26 63 7 16466176

[B104] Liu C Liu P Liu CH Zhu XQ Ji G Effects of Fuzhenghuayu decoction on collagen synthesis of cultured hepatic stellate cells, hepatocytes and fibroblasts in rats World J Gastroenterol 1998 4 548 549 11819368 10.3748/wjg.v4.i6.548 PMC4723451

[B105] Liu C Jiang CM Liu CH Liu P Hu YY Effect of Fuzhenghuayu decoction on vascular endothelial growth factor secretion in hepatic stellate cells Hepatobiliary Pancreat Dis Int 2002 1 207 10 14607740

[B106] Liu P Hu YY Liu C Xu LM Liu CH Sun KW Hu DC Yin YK Zhou XQ Wan MB Cai X Zhang ZQ Ye J Zhou RX He J Tang BZ Multicenter clinical study on Fuzhenghuayu capsule against liver fibrosis due to chronic hepatitis B World J Gastroenterol 2005 11 2892 9 15902724 10.3748/wjg.v11.i19.2892 PMC4305655

[B107] Liu CH Hu YY Xu LM Liu C Liu P Effect of Fuzheng Huayu formula and its actions against liver fibrosis Chin Med 2009 4 12 19558726 10.1186/1749-8546-4-12 PMC2720970

[B108] He Q Yang DG Li L Zhong BL Zeng XM The 24 week effectiveness of Fuzheng Huayu capsule for CHB liver fibrosis: a systematic assessment Zhongguo Xunzheng Yixue Zazhi 2008 8 892 7

[B109] Okuno T Arai K Shindo M Efficacy of interferon combined glycyrrhizin therapy in patients with chronic hepatitis C resistant to interferon therapy Nippon Rinsho 1994 52 1823 7 8078202

[B110] Tsubota A Kumada H Arase Y Chayama K Saitoh S Ikeda K Kobayashi M Suzuki Y Murashima N Combined ursodeoxycholic acid and glycyrrhizin therapy for chronic hepatitis C virus infection: a randomized controlled trial in 170 patients Eur J Gastroenterol Hepatol 1999 11 1077 83 10524635 10.1097/00042737-199910000-00002

[B111] Sun WH Song MQ Liu ZJ Combination therapy for hepatic fibrosis in 64 patients with hepatitis using Xiao-chai-hu-tang and Matrine injection Zhongxiyi Jiehe Ganbing Zazhi 2003 13 41 2

[B112] Li Z Liao HH Wu MJ Lin ZH Study of combination therapy of Interferon-gamma and Xiao-chai-hu-tang for patients with liver fibrosis Zhongxiyi Jiehe Ganbing Zazhi 2001 11 Suppl 95

[B113] Xiong F Sun J Xiong W Clinical observation of combination therapy of Interferon and Xiao-chai-hu-tang for patients with liver fibrosis Hubei Zhongyi Zazhi 2003 25 10 11

[B114] Nakagawa A Yamaguchi T Takao T Amano H Five cases of drug-induced pneumonitis due to Sho-saiko-to or interferon-alpha or both Nihon Kyobu Shikkan Gakkai Zasshi 1995 33 12 1361 1366 8821988

[B115] Ishizaki T Sasaki F Ameshima S Shiozaki K Takahashi H Abe Y Ito S Kuriyama M Nakai T Kitagawa M Pneumonitis during interferon and/or herbal drug therapy in patients with chronic active hepatitis Eur Respir J 1996 9 2691 6 8980988 10.1183/09031936.96.09122691

[B116] Liu AL Wei M Zeng ZG Sun JX Yang M Yan SY Combination therapy of Xiao-chai-hu-tang and Tiopronin for patients with liver fibrosis Shaanxi Zhongyi 2005 26 873 4

[B117] Chen YS The efficacy of combining Lamivudine with *Salvia miltiorrhiza *on the treatment of chronic hepatitis B liver fibrosis Redai Yixue Zazhi 2003 3 207 9

[B118] Chen JJ Tang BX Wang LT Chen XR Clinical study on effect of bushen granule combined with marine injection in treating chronic hepatitis B of Gan-shen deficiency with damp-heat syndrome type Zhongguo Zhongxiyi Jiehe Zazhi 2006 26 23 7 16466167

[B119] Liu X Hu H Yin JQ Therapeutic strategies against TGF-beta signalling pathway in hepatic fibrosis Liver Int 2006 26 8 22 16420505 10.1111/j.1478-3231.2005.01192.x

[B120] Choi HS Savard CE Choi JW Kuver R Lee SP Paclitaxel interrupts TGF-beta1 signaling between gallbladder epithelial cells and myofibroblasts J Surg Res 2007 141 183 91 17574589 10.1016/j.jss.2006.12.558 PMC3571727

[B121] Elsharkawy AM Oakley F Mann DA The role and regulation of hepatic stellate cell apoptosis in reversal of liver fibrosis Apoptosis 2005 10 927 39 16151628 10.1007/s10495-005-1055-4

[B122] Stickel F Patsenker E Schuppan D Herbal hepatotoxicity J Hepatol 2005 43 901 10 16171893 10.1016/j.jhep.2005.08.002

[B123] Ishii M Miyazaki Y Yamamoto T Miura M Ueno Y Takahashi T Toyota T A case of drug-induced ductopenia resulting in fatal biliary cirrhosis Liver 1993 13 227 31 8377599 10.1111/j.1600-0676.1993.tb00635.x

[B124] Yuen MF Tam S Fung J Wong DK Wong BC Lai CL Traditional Chinese medicine causing hepatotoxicity in patients with chronic hepatitis B infection: a 1-year prospective study Aliment Pharmacol Ther 2006 24 1179 86 17014576 10.1111/j.1365-2036.2006.03111.x

[B125] Feng Y Basic and Clinical Toxicology of Chinese Medicines 2009 Hong Kong: Commercial Press in press

[B126] Levy C Seeff LD Lindor KD Use of herbal supplements for chronic liver diseases Clin Gastroenterol Hepatol 2004 2 947 56 15551246 10.1016/S1542-3565(04)00455-0

[B127] Dhiman RK Chawla YK Herbal medicines for liver diseases Dig Dis Sci 2005 50 1807 12 16187178 10.1007/s10620-005-2942-9

[B128] Ye X Feng Y Tong Y Ng KM Tsao SW Lau GKK Sze C Zhang Y Tang J Shen J Kobayashi S Hepatoprotective effects of Coptidis rhizoma aqueous extract on carbon tetrachloride-induced acute liver hepatotoxicity in rats J Ethnopharmacol 2009 124 130 6 10.1016/j.jep.2009.04.003 19536921

[B129] Feng Y Luo WQ Zhu SQ Explore new clinical application of Huanglian and corresponding compound prescriptions from their traditional use Zhongguo Zhongyao Zazhi 2008 33 1221 5 18720877

[B130] Feng Y Siu K Wang N Ng KM Tsao SW Nagamatsu T Tong Y Bear bile: dilemma of traditional medicinal use and animal protection J Ethnobiol Ethnomed 2009 5 2 10.1186/1746-4269-5-2 19138420 PMC2630947

[B131] Zhao ZZ Hu YN Liang ZT Yuen JPS Jian ZH Lueng KSY Authentication is fundamental for standardization of Chinese Medicines Planta Medica 2006 72 1 10 10.1055/s-2006-947209 16902852

[B132] Geerts A Rogiers V Sho-saiko-to the right blend of Traditional oriental medicine and liver cell biology Hepatology 1999 29 282 3 9862880 10.1002/hep.510290129

[B133] Angell M Kassirer JP Alternative medicine-the risks of untested and unregulated remedies (Editorial) N Engl J Med 1998 339 839 41 9738094 10.1056/NEJM199809173391210

